# SMRT sequencing revealed the diversity and characteristics of defective interfering RNAs in influenza A (H7N9) virus infection

**DOI:** 10.1080/22221751.2019.1611346

**Published:** 2019-05-14

**Authors:** Wing-Yu Lui, Chun-Kit Yuen, Can Li, Wan Man Wong, Pak-Yin Lui, Chi-Ho Lin, Kwok-Hung Chan, Hanjun Zhao, Honglin Chen, Kelvin K. W. To, Anna J. X Zhang, Kwok-Yung Yuen, Kin-Hang Kok

**Affiliations:** a Department of Microbiology, Li Ka Shing Faculty of Medicine, University of Hong Kong, Hong Kong, People’s Republic of China; b Center for Genome Sciences, Li Ka Shing Faculty of Medicine, University of Hong Kong, Hong Kong, People’s Republic of China; c State Key Laboratory for Emerging Infectious Diseases, Li Ka Shing Faculty of Medicine, University of Hong Kong, Hong Kong, People’s Republic of China; d Carol Yu Centre for Infection, Li Ka Shing Faculty of Medicine, University of Hong Kong, Hong Kong, People’s Republic of China; e Collaborative Innovation Center for Diagnosis and Treatment of Infectious Diseases, Li Ka Shing Faculty of Medicine, University of Hong Kong, Hong Kong, People’s Republic of China

**Keywords:** Avian influenza A/H7N9 virus, defective interfering viral genome, Single Molecule Real Time sequencing, Illumina sequencing

## Abstract

Influenza defective interfering (DI) particles are replication-incompetent viruses carrying large internal deletion in the genome. The loss of essential genetic information causes abortive viral replication, which can be rescued by co-infection with a helper virus that possesses an intact genome. Despite reports of DI particles present in seasonal influenza A H1N1 infections, their existence in human infections by the avian influenza A viruses, such as H7N9, has not been studied. Here we report the ubiquitous presence of DI-RNAs in nasopharyngeal aspirates of H7N9-infected patients. Single Molecule Real Time (SMRT) sequencing was first applied and long-read sequencing analysis showed that a variety of H7N9 DI-RNA species were present in the patient samples and human bronchial epithelial cells. In several abundantly expressed DI-RNA species, long overlapping sequences have been identified around at the breakpoint region and the other side of deleted region. Influenza DI-RNA is known as a defective viral RNA with single large internal deletion. Beneficial to the long-read property of SMRT sequencing, double and triple internal deletions were identified in half of the DI-RNA species. In addition, we examined the expression of DI-RNAs in mice infected with sublethal dose of H7N9 virus at different time points. Interestingly, DI-RNAs were abundantly expressed as early as day 2 post-infection. Taken together, we reveal the diversity and characteristics of DI-RNAs found in H7N9-infected patients, cells and animals. Further investigations on this overwhelming generation of DI-RNA may provide important insights into the understanding of H7N9 viral replication and pathogenesis.

## Introduction

Influenza defective interfering (DI) particles are defective virions that harbour large internal deletions in at least one segment of the viral genome [1]. This loss of essential genetic information, mostly found in the three polymerase genes, renders these virions non-replicative, which can be reversed by co-infection with an intact helper virus [1,2]. DI particles are described as “interfering” because of their hindrance to viral replication and packaging of the co-infected helper virus [3]. Influenza DI-RNAs have been observed in embryonated chicken eggs and cell cultures with a serial passage of the virus at a high multiplicity of infection [4,5]. Generation of influenza DI-RNAs has been described in different strains of influenza infection, including H1N1 [4,6,7], H3N8 [8], avian H5N2 [9], avian H7N7 [10] and influenza B viruses [11]. Though the recently emerged H7N9 virus has caused severe human infections, little is known about the existence of H7N9 DI-RNAs.

High-throughput sequencing revolutionizes the field of DI particle research because of its ability to identify and quantitate different DI-RNA species. Recently, several studies attempt to use Illumina sequencing as a new approach for identifying different influenza DI species [11,12]. Unfortunately, short-read length Illumina sequencing may only provide partial information of individual DI species. Recently, the advancement in a long-read sequencing platform, Single Molecule Real Time (SMRT) sequencing, may overcome the existing barrier and allow further characterization of these DI species. SMRT sequencing is a third-generation sequencing technology that generates sequencing reads of extended length (>10 kb). Without the need of RNA fragmentation before sequencing and the later bioinformatic read assembly, SMRT sequencing allows precise discrimination of different alternatively spliced transcripts or isoforms [13]. Since DI-RNAs containing large internal deletion are analogous to alternatively spliced transcripts, SMRT sequencing can be an alternative approach to next generation sequencing (NGS) for determining the comprehensive information of DI-RNA species.

In this study, we observed the presence of DI-RNAs in nasopharyngeal aspirates (NPAs) from patients who were infected by H7N9 in high abundance and adopted SMRT sequencing to identify the entire sequence of these DI-RNA species. From the identified DI-RNA sequence, the highly diverse DI-RNA species generated in patients with H7N9 infection could be observed. Intra-species overlapping sequences at both ends of the large internal deletion around the putative breakpoint were noted. We have further extended our study of DI-RNA species in cell line and mouse model infected with H7N9 AH1 strain with SMRT sequencing. We also compared the identified DI-RNA species from infected mouse model with both Illumina and SMRT sequencing, and found that both platforms were able to identify the common large internal deletion breakpoint but SMRT could also detected DI-RNA species with multiple breakpoints owing to its long sequencing read length. While the exact mechanism of the generation of the DI-RNA by influenza has not been revealed, SMRT appears a promising tool for characterizing DI-RNA entities in future mechanistic studies.

## Materials and methods

### Virus preparation

Two influenza A viruses, A/WSN/1933 [H1N1] and A/Anhui/1/2013 [H7N9], were used in this study. H1N1 (WSN) virus was generated using pHH21-plasmid reverse genetics system [14]. H7N9 (A/Anhui/1/2013) has been described elsewhere [15]. The two viruses were propagated in 10-day-old specific pathogen-free embryonated chicken eggs at 37°C for 2 days. Viruses were plaque-purified and propagated in Madin Darby canine kidney (MDCK) cells. Viral titre (plaque forming units; PFU) was determined in MDCK cells by plaque assay as previously described [16].

### Human clinical samples

Testing of all patients’ specimens has been approved by the Institutional Review Board of the University of Hong Kong/Hospital Authority Hong Kong West Cluster (HKU/HA HKW IRB) (UW 13–372). Six NPAs of 10–76-year-old Chinese patients with H7N9 infection were collected in Hong Kong during 2014–2017 and cryo-preserved. NPAs of four Chinese patients with H3N2 infection were collected in Hong Kong during 2017.

### Cell culture

Human normal bronchial epithelial cells (NHBE) were purchased from Lonza (Cat# CC-2540). NHBE were cultured in 1:1 ratio of EpiLife^TM^ medium (Gibco Cat# MEPI500CA) supplemented with EpiLife^TM^ Defined Growth Supplement (EDGS) (Gibco Cat# S0125) and Keratinocyte-SFM Medium (Gibco Cat# 17005042), including the Keratinocyte Supplements which contain bovine pituitary extract and recombinant EGF.

### Virus infection

NHBE cells were seeded in six-well plates and infected with H1N1 or H7N9 at multiplicity of infection (MOI) of 0.5 for 1 h at 37°C supplemented with 5% CO_2_. Cells were then washed twice with blank RPMI 1640 and cultured in RPMI 1640 medium supplemented with TPCK-trypsin (Sigma). Total cellular RNA was extracted at 24 h post-infection.

### Animals and virus inoculation

All animal experiments were followed to the standard operating procedures approved by the University of Hong Kong committee on the use of live animals in teaching and research (CULATR: 3536-14). Six- to eight-week-old female BALB/c mice were obtained from the Laboratory Animal Unit of the University of Hong Kong. Virus challenge experiments were performed in biosafety level 2 (WSN virus) and 3 (H7N9 virus) animal facilities. Mouse 50% lethal dose (LD50) was determined with serial dilutions of H7N9 virus by intranasal inoculation as previously described [17]. The LD50 of H7N9 virus was determined 10^4.8^ PFU [18]. Group of 6–8-week-old female BALB/c mice were intranasally inoculated with 10^3^ PFU of H7N9 virus. The mouse lung tissues and homogenates were collected on 2 and 4 days after virus inoculation for the quantitation of DI-RNA.

### RNA extraction, RT-PCR and Sanger sequencing

Viral RNA was extracted from NPA of H7N9-infected patients and H7N9-infected NHBE using a QIAamp Viral RNA Mini kit (Qiagen) following the manufacturer’s instructions. Total RNA was extracted from virus-infected NHBE cells and mouse lung tissues using Trizol reagent (Invitrogen) following the manufacturer’s instructions. Random hexamer primers were used to synthesize cDNA from viral or total RNA using a Transcriptor First Strand cDNA Synthesis kit (Roche Molecular Systems, Inc). Primers designed for DNA amplification of PB1 DI-RNA detection in mice and SMRT sequencing were shown in Supplementary Table S1. The viral genome and total RNA were amplified using a PrimeSTAR® GXL DNA Polymerase (TaKaRa). PCR amplification cycled under the following condition: 95°C for 3 min, followed by 30 cycles of 98°C for 10 s, 60°C for 15 s and 68°C for 3 min. Following electrophoresis in 1% agarose gel and the relative intensity of DI-RNA and full length RNA was determined by ImageJ v1.52. All fragments within 100–1000 bp were considered as DI-RNA and fragments within 1500–2500 bp were classified as full length RNA.

To perform Sanger sequencing on the common DI-RNA isolated from the PB1 segment of H7N9-infected patient NPA sample, its PCR product was purified using a PureLink^TM^ Quick Gel Extraction Kit (Invitrogen). The gel-purified product was TA-cloned into pGEM-T vector using pGEM-T and pGEM-T Easy Vector Systems (Promega) which was then plasmid-extracted with PureLink^TM^ Quick Plasmid Miniprep Kit (Invitrogen) and subjected to Sanger sequencing. Sequences of eight individual clones were aligned to the full length PB1 sequence.

### Illumina sequencing

One of the H7N9-infected mice was selected for identifying DI-RNAs using Illumina sequencing. The Illumina sequencing and library construction was performed by Novogene. In brief, NEBNext® Ultra RNA Library Prep Kit was used for library construction. After adapter ligation, 10 cycle of PCR amplification was performed for sequencing target enrichment. Sequencing was performed with 150 bp paired end reads on an Illumina NovaSeq 6000. FASTQ sequences of each sample were aligned against the H7N9 (A/Anhui/1/2013) sequence using TopHat2 [19] with 5–100,000 nucleotides intron length allowed. Sequences with internal deletion were extracted and aligned against A/Anhui/1/2013 strain (EPI439503-5; EPI439508) limited to at least seven nucleotides at one side of the internal deleted sequences. The breakpoint position of these sequences was determined by the gap opening and closing position of the alignment by using in-house python script based on pairwise2 [20].

### SMRT sequencing

The remaining PCR products (NPAs of patient #3, #5, #6, and H1N1- and H7N9-infected NHBE or mice samples) were cleaned up using GeneJET Gel Extraction and DNA Cleanup Micro Kit (Thermo Fisher). The second round DNA amplification was performed using KAPA HiFi HotStart PCR kit (Roche) with Barcoded Universal Primers (Pacific Biosciences). The barcoded PCR products were pooled for the SMRTbell library construction using SMRTbell^TM^ Template PreP Kit 1.9-SPv3. One Sequel SMRT cell was used to sequence 7 amplicons. The raw reads were separated and circular consensus sequence (CCS) reads were generated using SMRT Link v5.0.1. The SMRTbell adapter sequences were removed and CCS reads within 150–2400 bp were selected for further analysis. The histograms of numbers of counts and length of DI-RNA sequence were generated using R [21]. The sequencing data of H7N9-infected NPAs (#3, #5 and #6) and NHBE in Supplementary Table S3 was analysed as followed. Sequences were aligned against the reference genome of H7N9 A/Anhui/1/2013 strain (EPI439503-5; EPI439508) using NCBI blastn alignment tools to determine the position of breakpoint. The identity of a single DI-RNA species was established by the identical sequence of 40–100 nucleotides long flanking the breakpoint of DI-RNA. The sequencing data of the remaining samples (H7N9-infected mouse in Supplementary Table S3 and samples in Supplementary Table S2) was analysed as followed. Sequences were first aligned against the reference genome of H7N9 A/Anhui/1/2013 strain (EPI439503-5; EPI439508) and the breakpoint position of internal deleted sequences was determined using in-house python script that used in Illumina sequencing. Read counts of the identical DI-RNA species were calculated to determine the abundancy of DI-RNA species.

## Results

**Figure 1.**
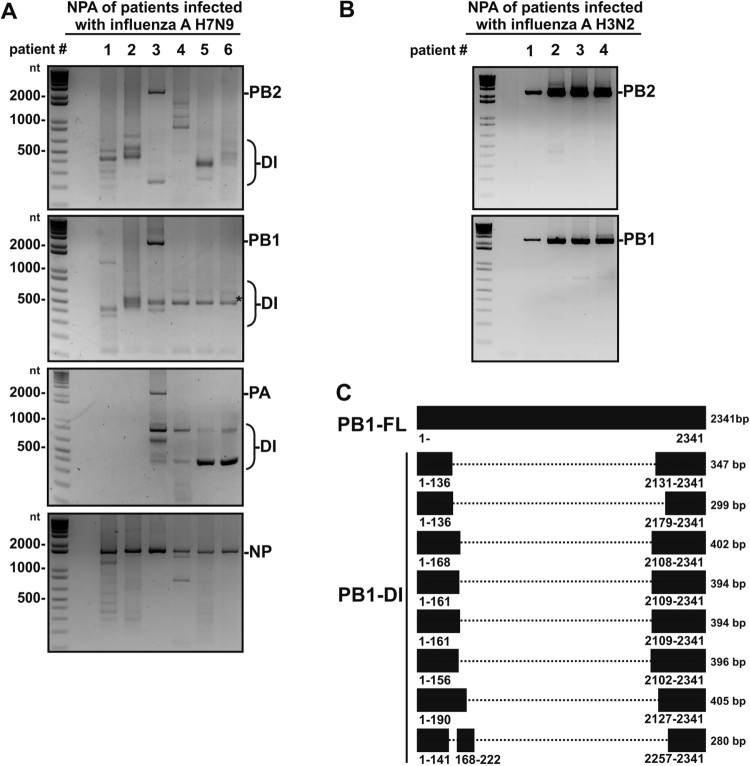
Identification of defective interfering RNA (DI-RNAs) in NPAs of H7N9-infected patients by Sanger sequencing. (A) DI-RNAs were detected in the NPAs of six patients infected with influenza A H7N9 but (B) unable to be detected in NPAs of patients infected with influenza A H3N2. Viral RNAs were extracted from the NPA samples and DI-RNAs were visualized by semi-quantitative RT-PCR and gel electrophoresis. Asterisk (*) indicates one of the abundantly expressed PB1 DI-RNAs commonly detected in five out of six NPA samples. (C) Sequences of DI-RNAs were determined by Sanger sequencing. The PCR products of PB1 derived from the samples of patient #6 were subcloned into TA cloning vectors and subjected to Sanger sequencing. Dotted lines represent internally deleted regions of DI-RNAs. nt, nucleotide; bp, base pair; FL, full length RNA; DI, defective interfering RNA.

DI-RNAs are most frequently identified in polymerase genes PB2, PB1, and PA, but not in nucleoprotein NP [22]. Therefore, we mainly focused on characterizing DI-RNAs of the three polymerase genes, with inclusion of NP gene as a negative control. Six NPAs of 10–76-year-old Chinese patients with H7N9 infection were collected in the third and fifth waves of H7N9 influenza epidemic. Among these six patients, two patients were scored as critically ill and two result in death after H7N9 infection. The details of potential poultry exposure and severity of illness among these six Chinese patients were listed in Table 1. In these NPA, multiple DI-RNA species in the range from 200 to 800 bp could be detected by RT-PCR in PB2, PB1 and PA segments (Figure 1A). No correlation was observed between the production of DI-RNAs and severity of illness among these six patients. To demonstrate RT-PCR technique may less likely result in generation of defective genomic cDNAs, NPAs of four patients with H3N2 infection were collected during 2017 in Hong Kong and were used as the controls. A very strong signal of full length PB1 and PB2 was observed in these four NPA samples but very low or no signal for DI-RNAs (Figure 1B). Compared to the NPA of H3N2-infected patients, the expression of DI-RNAs was relatively higher in H7N9-infected patient’s NPAs. To confirm that these DI-RNAs contained internal deletions, one of the consistently detected PB1 DI-RNAs from NPA of H7N9-infected patient #6 (indicated with an asterisk in Figure 1A) was purified and Sanger-sequenced. To our surprise, seven DI-RNA species from eight sequenced plasmid clones were detected, exemplified by one DI-RNA containing a single large internal deletion and another containing two consecutive internal deletions (Figure 1C).

**Table 1.** Severity of illness and poultry exposure of six Chinese patients with H7N9 infection during 2014–2017 (in the third- and fifth-wave H7N9 influenza A viruses).

Patient no.	Age	Gender	Location	Year	Wave	Severity	Days of sample collection after symptom onset	Poultry exposure	SMRT sequencing
1	75	M	Hong Kong	2016	5	Death	2	Bought slaughtered chicken from wet market and cooked it at home	
2	70	M	Hong Kong	2016	5	Stable	3	Saw mobile stalls selling live poultry but denied contact	
3	10	M	Hong Kong	2017	5	Stable	Not determined	Visited wet market; stayed relatives’ home where live chicken were raised	In Figure 3
4	68	F	Hong Kong	2014	3	Critical	Not determined	Visited wet market in Shenzhen and saw chicken stall within 2–3 feet	
5	61	M	Hong Kong	2015	3	Death	7	Visited wet market in Dongguan and bought slaughtered chickens	InFigure 3
6	76	M	Hong Kong	2017	5	Critical	4	Bought slaughtered mutton from wet market in Fuzhou	InFigure 3

**Figure 2.**
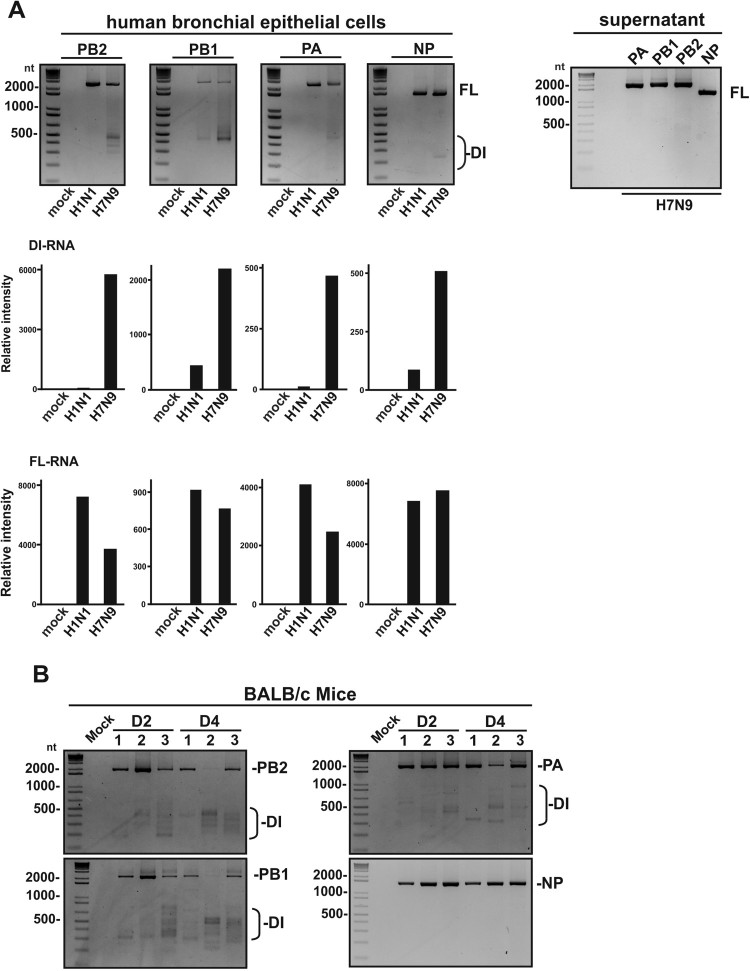
Detection of DI-RNAs in H7N9-infected human bronchial epithelial cells (NHBEs). (A) NHBEs were infected with H7N9 (A/Anhui/1//2013) and H1N1 (A/WSN/1933) at multiplicity of infection of 0.5 for 24 h. Total RNA was extracted from the H7N9-infected NHBE and DI-RNAs were visualized by semi-quantitative RT-PCR and gel electrophoresis. The relative intensity of DI-RNA and full length RNA was determined by ImageJ v1.52. All fragments within 100–1000 bp were considered as DI-RNA and fragments within 1500–2500 bp were classified as full length RNA. Two replicates of PCR from a single experiment were performed. (B) Detection of DI-RNA in H7N9-infected mice. Six- to eight-week-old female BALB/c mice were challenged intranasally with 10^3^ PFU of H7N9 for 2 and 4 days (*n* = 3). Total RNA of H7N9-infected mice were extracted and followed by RT-PCR. nt, nucleotide; bp, base pair; FL, full length RNA; DI, defective interfering RNA.

Because of abundant DI-RNAs found in the NPAs of H7N9-infected patients, one of the reference H7N9 strain (A/Anhui/1/2013 – AH1) was selected for examining the generation of DI-RNAs in cell culture and mouse model. Since H1N1 WSN strain has been reported to generate DI-RNAs in infected cultured cells with available sequence information [23], we compared H7N9 (A/Anhui/1/2013) with H1N1 (WSN) for their ability to produce DI-RNAs in NHBEs during infection. H1N1 (WSN) and H7N9 (AH1) virus stocks used in NBHE and mice infection were confirmed without any DI-RNA present before mice and NHBE infection (Supplementary Figure S1). Since the DI-RNAs are well known to be produced in undiluted passage of H1N1 (WSN) viruses or with high MOI infection, low MOI infection was performed to demonstrate the robustness of DI-RNA generation by H7N9 (AH1) virus. Due to a relatively low MOI infection (MOI = 0.5), insignificant amount of DI-RNAs was detected in NHBEs with H1N1 (WSN) infection. On the contrary, copious DI-RNAs from PB1 and PB2 segments and, to a lesser extent, from PA segment, were detected in H7N9-infected cells; however, DI-RNA was not observed in the supernatant virions (Figure 2A). This contrasting observation may imply that H7N9 (AH1) has greater potential in generating DI-RNAs at a lower MOI infection. Among different MOI infections (MOI = 0.01, 0.1 and 0.5) of H7N9 (AH1) virus, the production of DI-RNA can only be observed in the NHBE cells with 0.5 MOI H7N9 (AH1) infection (data not shown). In addition to examine the DI-RNA formation in H7N9-infected NHBE cells, BALB/c mice were also used as a model to determine the presence of DI-RNA with the infection of H7N9 (AH1). Female BALB/c mice were intranasally inoculated with 10^3^ PFU of H7N9 (AH1) viruses. High amount of DI-RNA was detected in H7N9-infected lung tissues as early as days 2 and 4 post-infection (Figure 2B). The patient’s NPA with H7N9 infection (the third and fifth epidemic waves), H7N9 (AH1) infected NHBE cells and mice suggested the existence of DI-RNA during influenza A (H7N9) virus infection.

To gain a more comprehensive understanding on the variety of DI-RNA species, high-throughput sequencing was applied to detect and quantity the abundant of different DI species. Currently, the sequencing platform used for identifying internal deletion of influenza genome is Illumina sequencing [11,24]. As the rise of the third-generation sequencing, the sequencing read length was improved tremendously and may increase the amount of information obtained from the sequencing. Therefore, we selected one of the H7N9-infected mice sample for the comparison of both sequencing platforms – paired end 150 bp Illumina sequencing and SMRT sequencing in this study. The raw reads of SMRT and Illumina sequencing of H7N9-infected mice were listed in Supplementary Table S2. Due to requirement of amplification in SMRT sequencing, higher depth of coverage was achieved from SMRT sequencing, leading to more aligned DI-RNA sequences and unique DI-RNA species were obtained compared to Illumina sequencing (Table 2). Moreover, the abundant DI-RNA species identified from Illumina sequencing can also be found in SMRT sequencing, such as PA DI-492, PB1 DI-675 and PB2 DI-376 (highlighted as blue in the Supplementary Table S3). Most importantly, multiple internal deletions of DI-RNAs can be identified using the long-read length SMRT sequencing, which were unable to achieve using Illumina sequencing. Within all the DI-RNAs obtained from Illumina sequencing, no multiple internal deletion DI-RNA was identified, including PA DI-492 and PB1 DI-675 that were defined as double internal deletion DI-RNA in SMRT sequencing.

**Table 2.** Comparison of sequencing result of H7N9 (AH1) infected mouse sample by Illumina and SMRT sequencing.

	Illumina sequencing	SMRT sequencing (CCS)
Total number of bases	4,952,373,600	127,368,605
Total number of reads aligned to influenza	298,723	143,896
Number of reads for PB2, PB1, PA, and NP	203,719	143,896
Number of reads for each segment	PB2: 37,406 PB1: 96,493 PA: 44,477 NP: 25,343	PB2: 56,020 PB1: 41,035 PA: 22,410 NP: 18,094
Average depth of coverage	3726	15,020
Average read length	150	885
Longest read length	150	6888
GC content	50.58%	45.80%
Aligned DI sequences (read count)	593	110,606
Single internal deletion (read count)	593	56,153
Double internal deletion (read count)	0	40,435
Triple internal deletion (read count)	0	9970
Number of unique DI-RNA species	220	7945

**Figure 3.**
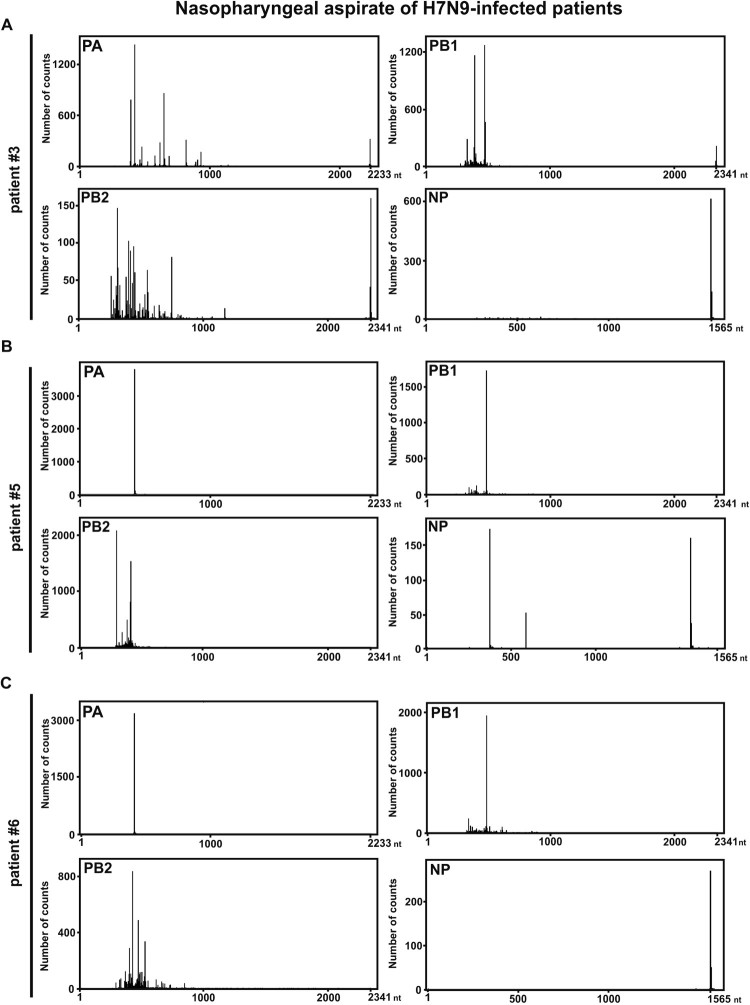
SMRT sequencing of NPAs of three reported patients with H7N9 infection. Viral RNAs were extracted from the NPAs of (A) #3, (B) #5 and (C) #6 patients with influenza A H7N9 infection. Four segments (PA, PB1, PB2 and NP) were sequenced by SMRT sequencing to determine the abundancy of DI-RNAs. The histograms depict the size distribution of DI-RNAs from PA, PB1, PB2, and NP segments. The sequences of identified DI-RNA species were listed in Supplementary Table S3.

**Figure 4.**
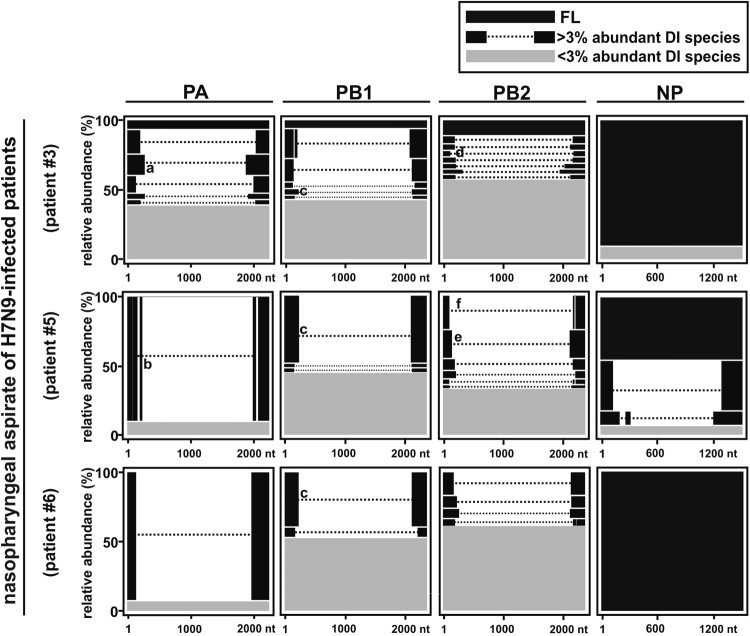
Abundance of DI-RNAs in NPAs of three H7N9-infected patients determined by SMRT sequencing. RNAs were extracted from the NPAs of three H7N9-infected patients and subjected to long-read SMRT sequencing. The most abundantly expressed DI-RNA species as well as the full length RNA in PA, PB1, PB2, and NP segments were presented in relative abundance (%). Black bars represent the full length influenza RNAs and DI-RNA species with more than 3% relative abundancy of PA, PB1, PB2, and NP segments. Grey bars represent the remaining DI-RNA species that were less than 3% relative abundancy. Dotted lines represent internal deletions. Sequences of DI-RNAs were listed in Supplementary Table S3. The DI-RNA species with longer overlapping sequences around breakpoint region were indicated (a–b, d–f) and details are showed in Figure 7.

SMRT sequencing was applied for the following experiments due to the identification of rare DI-RNA species and receiving more comprehensive information. The raw reads of SMRT sequencing for all H7N9-infected individuals were listed in Supplementary Table S2. The three NPAs of H7N9-infected patients with the highest sample quality and viruses-infected NHBEs were selected and subjected to SMRT sequencing. Consistent with the DI-RNA pattern observed in gel electrophoresis, high abundance of DI-RNAs in PA, PB1 and PB2 was revealed in SMRT sequencing but relatively less abundant for NP segment (Figure 3). In-depth sequence analysis on breakpoint identification suggested that the SMRT long-read sequencing could precisely display the diversity and relative abundance of DI-RNAs species found in the three patient-derived NPAs (Figure 3 and Supplementary Table 2). As representative, the three most abundant DI-RNA species of the PA, PB1, PB2 and NP genes from each NPA sample were selected. The internal deletion of these abundantly expressed DI-RNAs was graphically illustrated in Figure 4. The sequence information including break point positions and number of counts was detailed in Supplementary Table 2. Using this long-read sequencing approach, we were able to identify various DI-RNA species with single, double or even multiple internal deletions in the NPA of H7N9-infected patients (Figure 4 and Supplementary Table 2). Most of these double or triple internal deletions consist of less than 20 nt deletions before the large internal deletion. Five out of seven DI-RNA species found in NPA of H7N9-infected patient #6 using Sanger sequencing could also be identified by using SMRT sequencing. Among the seven DI-RNA species detected by Sanger sequencing, PB1 2109 and 2127 DI-RNA species were the only DI-RNA species that obtained more than 10 reads in SMRT sequencing (Supplementary Table 2); only one or two reads were obtained for DI-RNA species detected by Sanger sequencing.

**Figure 5.**
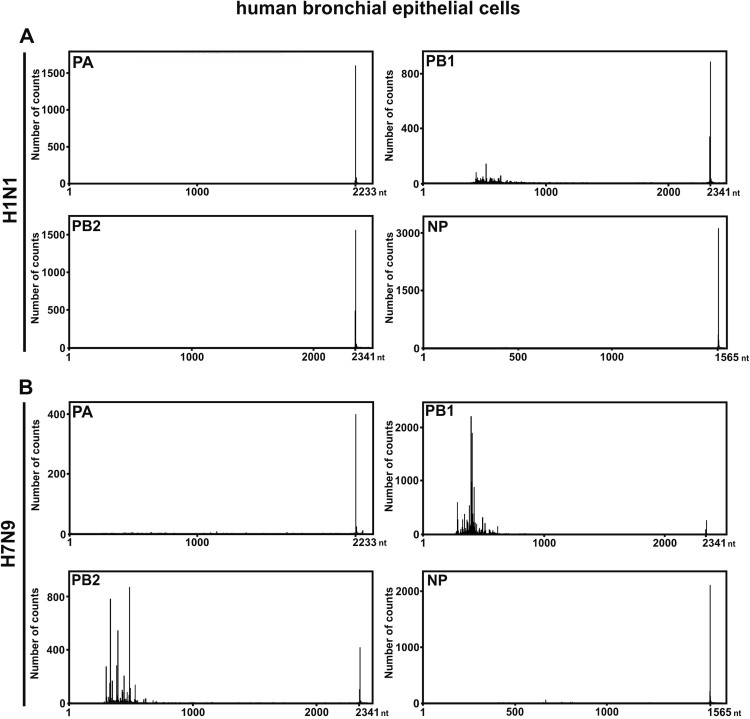
SMRT sequencing of human bronchial epithelial cells with (A) H1N1 or (B) H7N9 infection. Total RNAs were extracted from H1N1 (A/WSN/1933)- or H7N9 (A/Anhui/1//2013)-infected human bronchial epithelial cells and subjected to SMRT sequencing. The histograms depict the size distribution of DI-RNAs in PA, PB1, PB2, and NP segments. The sequences were listed in Supplementary Table S3.

**Figure 6.**
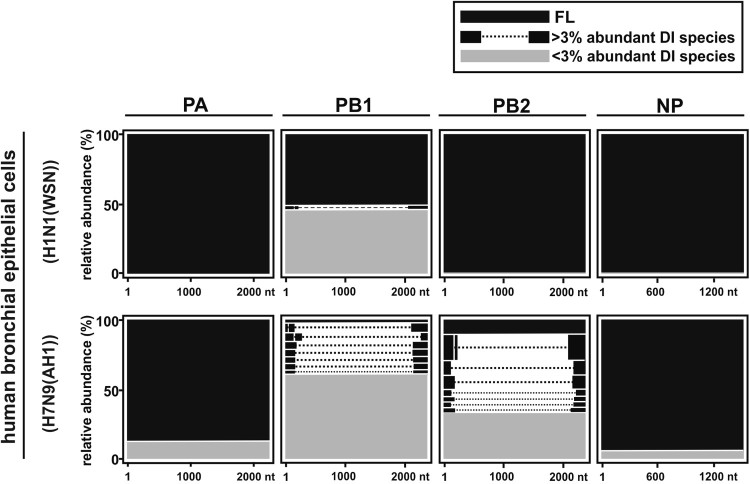
Abundance of DI-RNAs in H1N1 and H7N9-infected NHBE determined by SMRT sequencing. Total RNAs were extracted from H1N1 (A/WSN/1933)- or H7N9 (A/Anhui/1//2013)-infected human bronchial epithelial cells and subjected to SMRT sequencing. The most abundantly expressed DI-RNA species as well as the full length RNA in PA, PB1, PB2, and NP segments were presented in relative abundance (%). Black bars represent the full length influenza RNAs and DI-RNA species with more than 3% relative abundancy of PA, PB1, PB2, and NP segments. Grey bars represent the remaining DI-RNA species that were less than 3% relative abundancy. Dotted lines represent internal deletions. Sequences of DI-RNAs were listed in Supplementary Table S3.

**Figure 7.**
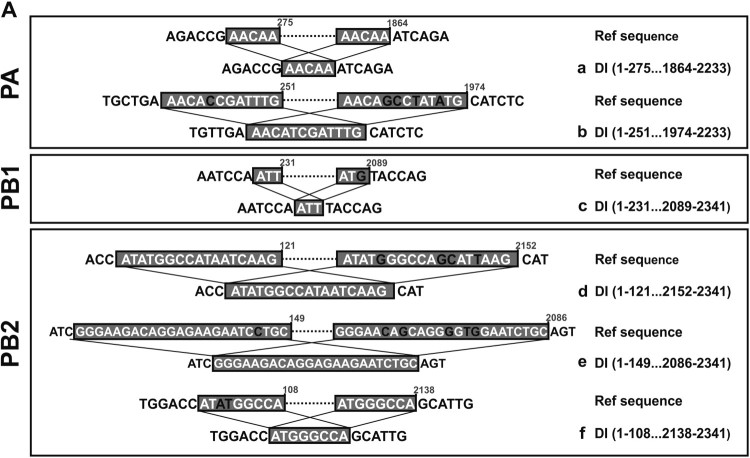
Characterized overlapping region of selected DI-RNA of PA, PB1, and PB2 segments from NPAs of patients #3, #5, and #6. Longer overlapping sequences (ranged 5–23 bp) in PA (a and b) and PB2 segment (d–f) were found in the abundant DI-RNA species. Shorter overlapping sequencing was listed in Supplementary Table S3. Boxed sequences are overlapping sequences. Inconsistent or missed nucleotides are highlighted as black. nt, nucleotide; FL, full length RNA; DI, defective interfering RNA.

After aligning DI-RNA sequences to the referenced sequences, similar observation was invariably resulted from SMRT sequencing of H7N9-infected NHBEs while DI-RNA was minimally detected in H1N1-infected cells by contrast (Figure 5 and Supplementary Table 2). The three representative DI-RNA species of PA, PB1, PB2, and NP genes were showed in Figure 6. In line with the published sequences of H1N1 DI-RNAs, no conserved motifs at the breakpoint sites could be observed among different H7N9 DI-RNA species. However, the identical breakpoints at 2109 and 2089 nt in PB1 segment could be found in all three patient’s NPA and H7N9-infected NHBEs (Supplementary Table 2; highlighted in green). In addition to identical breakpoints founded in PB1 segment of the three clinical specimens, sequences before the breakpoint sites were similar or identical to the deleted sequences on other side of the breakpoint sites (previously described as overlapping sequences [7]). Overlapping sequences (ranged from 2 to 23 nt) were observed around the breakpoint region of several DI-RNAs (Supplementary Table 2). As representative, longer overlapping sequences (ranged from 5 to 23 bp) in the three patient’s NPA and the identical PB1 DI-RNA species found in the clinical specimens graphically illustrated in Figure 7. Longer overlapping region (around 5–23 bp) was identified at the breakpoint of some of the most abundant DI-RNA species of PB2 and PA (Figure 7), which has not been previously reported in the literature.

## Discussion

This study demonstrated the robustness of SMRT sequencing in identifying the DI-RNA species. SMRT sequencing could successfully demonstrate the diversity of DI-RNA species and the relative abundance of each species. With the long-read length of SMRT sequencing, complete information of each DI-RNA could be obtained due to no fragmentation of DI genome required. For example, long overlapping sequences at the breakpoint region, and most importantly, DI-RNAs with multiple internal deletions were identified. In addition, the influenza DI-RNAs have been reported in cultured cells with influenza B virus infection [11] and H1N1-infected cultured cells [4], mice [25] and patient’s NPA [7,12]; however, the DI-RNA existence in human infections with avian influenza A H7N9 virus has not been revealed. This study provides evidence on the existence of DI-RNA in the clinical specimens, cultured cells and mice during influenza A (H7N9) virus infection. However, whether the production of DI-RNA can be generalized to all influenza A (H7N9) virus warrants further investigation since a robust production of H7N9-infected clinical specimens was observed, but H7N9 (AH1) used in this study is a low-pathogenic (LPAI) influenza A virus. It will be especially important to investigate and compare the DI-RNA production by both LPAI and high-pathogenic (HPAI) H7N9 influenza A virus.

The introduction of Sanger sequencing revolutionizes the field of DI particles due to identification of the breakpoint location for a single DI genome. Since then, Sanger sequencing becomes a traditional and standard sequencing platform for detecting and identifying DI-RNA species. Recently, NGS has been introduced and several studies attempt to use this new approach for identifying different DI species [7,11,12]. Apart from accessing the genomic information of a single DI genome, multiple DI species and their abundance can be determined by a single run of Illumina sequencing. Recently, a long-read sequencing platform – SMRT sequencing showed high quality and promising sequencing results that may also feasible for identifying DI-RNA species in clinical specimens. The comparison of Illumina and SMRT sequencing was performed before using SMRT sequencing as further investigation on DI-RNA species in clinical specimens. Our results demonstrated that both SMRT and Illumina sequencing can identify and showed the diversity of DI-RNA species in H7N9 (AH1)-infected mouse. Especially, the DI-RNA species with high abundance detected in SMRT sequencing were also abundantly found in Illumina sequencing. However, higher read counts and more comprehensive information of DI-RNAs were received from SMRT sequencing. As the result, SMRT sequencing was used for the further investigation on DI-RNA species in clinical specimens and infected NHBE cells. Detailed comparison between SMRT and Illumina sequencing was listed in Table 3. In brief, with the generation of a single-stranded circular DNA, a longer read length sequencing allows clear identification of DI breakpoint position without fragmentation of DI genome. Moreover, SMRT sequencing can also determine the relative abundance of DI species because each small chamber on SMRT chip associates with a single DNA entity. Our sequencing data demonstrate the robustness of SMRT sequencing as an alternative sequencing platform for detecting multiple DI-RNA species. Taken together, SMRT sequencing allows us to comprehensively assess the identity, abundance, and variety of various DI-RNA species present in patient-derived NPAs and infected cultured cells by avian H7N9 virus.

**Table 3.** Technical comparison of Illumina and SMRT sequencing.

	SMRT sequencing	Illumina sequencing
Sequence directly from NPA	Both can directly sequence from clinical specimens	
Multiplex	Up to 96 samples (defined by PacBio)	Also allows multiplex; Special oligonucleotides had already designed by others
Read length	Long; can sequence the full length of each influenza segments	Short; 150 bp paired reads in this study
Fragmentation	Not required	Required
PCR amplification	Two rounds of PCR amplification	Up to 12 cycles PCR amplification
Diversity of DI-RNA species detected	Can detect rare DI-RNA species	Only can detect very abundant DI-RNA species
Ratio between DI-RNA and full length segments	Can be precisely determined	Can be roughly determined

With the ability to obtain long-read length by SMRT sequencing, the comprehensive information of DI-RNA species could be obtained from both H7N9-infected clinical specimens and primary culture cells. From the high-throughput sequencing data of the three clinical NPA samples, it was observed that neither of these samples has identical DI formation pattern in any of the polymerase genes, apart from a few conserved DI patterns that was identified in multiple samples. Given that the current understanding in the formation of influenza DI-RNA is limited, the exact mechanism leading to the diversity of DI-RNA species production is still unknown. It could be possible that either the polymerase complex initiates the generation of DI-RNA in a random manner or the molecular difference of the sequence in the internal zgenes of these patients could give rise to this observation. With the feasibility to identify exact DI species present with influenza infection in cultured cell system, the molecular mechanism of DI-RNA generation could be elucidated with bioinformatics analysis of SMRT-sequenced *in-vitro* infection experiment in further studies.

In addition to the identification of multiple DI-RNA species in SMRT sequencing, similar or identical sequences are shown around the breakpoint region and the other side of deleted region (previously described as overlapping sequences [7]), which has been described previously [7]. According to those studies reported previously, the length of these overlapping sequences is often around 2–6-nt long in H1N1-infected individuals [7,26]. In this study, apart from short similar or identical overlapping sequences, longer overlapping sequences with up to 23-nt long were also observed around the breakpoint of several abundantly expressed DI-RNA species. These overlapping sequences may indicate that the translocation of viral polymerase possibly occurs during influenza A virus replication. Although the molecular mechanism of DI-RNA generation is still unknown, several reports showed increased accumulation of DI-RNA with mutations in PA [12,27] and NS segments [28,29]. Recently, a report suggested that polymerase PA D529N mutation led to the reduction of DI-RNA production during H1N1 infection [12]. However, PA D529N mutation is absent in our tested samples and other commonly used H7N9 reference strains. A comparative study of overlapping sequences of DI-RNA by SMRT sequencing with the amino acid substitution in different segments may provide insights for further investigation on the mechanism of influenza DI-RNA generation.

Robust expression of DI-RNA was observed in the H7N9-infected clinical specimens, cell culture and mice. Recent study suggested that the reduction of DI-RNA production in H1N1 viruses led to low antiviral response induction and increased viral pathogenesis [12]. Unfortunately, no correlation between the severity of illness and DI-RNA expression was observed in the six Chinese patient’s NPA with H7N9 infection. Due to insufficient sample number, further investigation on any correlation between the presence of DI-RNA and the severity of illness is required. Because the robust expression of DI-RNA was observed in H7N9-infected patients, we also identified the presence of DI-RNA in NHBE cells and mice with H7N9 infection. Unfortunately, the production of DI-RNA was not observed in the supernatant virions, which might be due to the low MOI infection. In previous reports [1,30], H1N1 (WSN) DI-RNAs were discovered in undiluted passage of H1N1 (WSN) viruses or with high MOI infection. To demonstrate the robustness of DI-RNA generation by H7N9 (AH1) virus, a low MOI infection of H1N1 (WSN) or H7N9 (AH1) was performed in NHBE cells. An insufficient amount of DI-RNAs in H1N1-infected NHBE cells was observed due to the low MOI infection. The DI-RNA production was significantly higher in NHBE with H7N9 (AH1) infection compared to H1N1 (WSN) infection. Moreover, H1N1 (WSN) DI-RNAs were previously reported in the mice lung [30] and DI viruses [5] generated from MDCK cells. However, the H1N1 (WSN) DI-RNA species that reported previously were unable to identify in this study. Therefore, it is possible that low MOI infection may give rise to other DI-RNA species and further investigation of comparing DI-RNA species in low MOI and high MOI infection is required.

In conclusion, our data demonstrates the existence of DI-RNAs in clinical specimens and cultured cells and mice model during influenza A (H7N9) virus infection. We also show that SMRT sequencing is a promising alternative sequencing technique which provides comprehensive genetic information and relative abundance of multiple DI-RNA species. Using the third-generation sequencing, we identify multiple abundant DI-RNA species in different clinical specimens and their overlapping sequences at the breakpoint regions. Increased DI-RNA synthesis is observed in H7N9-infected NHBE cells compared to the reported H1N1 (WSN) strain that generates DI-RNAs. All the studies mentioned above revealed the overwhelming synthesis of DI-RNAs during infection of H7N9 influenza A virus.

## Accession number(s)

The raw data of the SMRT sequencing reads were submitted to Genbank under Genbank accession number SRP126530.

## Supplementary Material

Supplemental Material
