# Supplementary figures and images for: SMRT sequencing revealed the diversity and characteristics of defective interfering RNAs in influenza A (H7N9) virus infection
Source: Emerg Microbes Infect. 2019 May 14;8(1):662–74. doi: 10.1080/22221751.2019.1611346 (PMC6534226; doi:10.1080/22221751.2019.1611346)

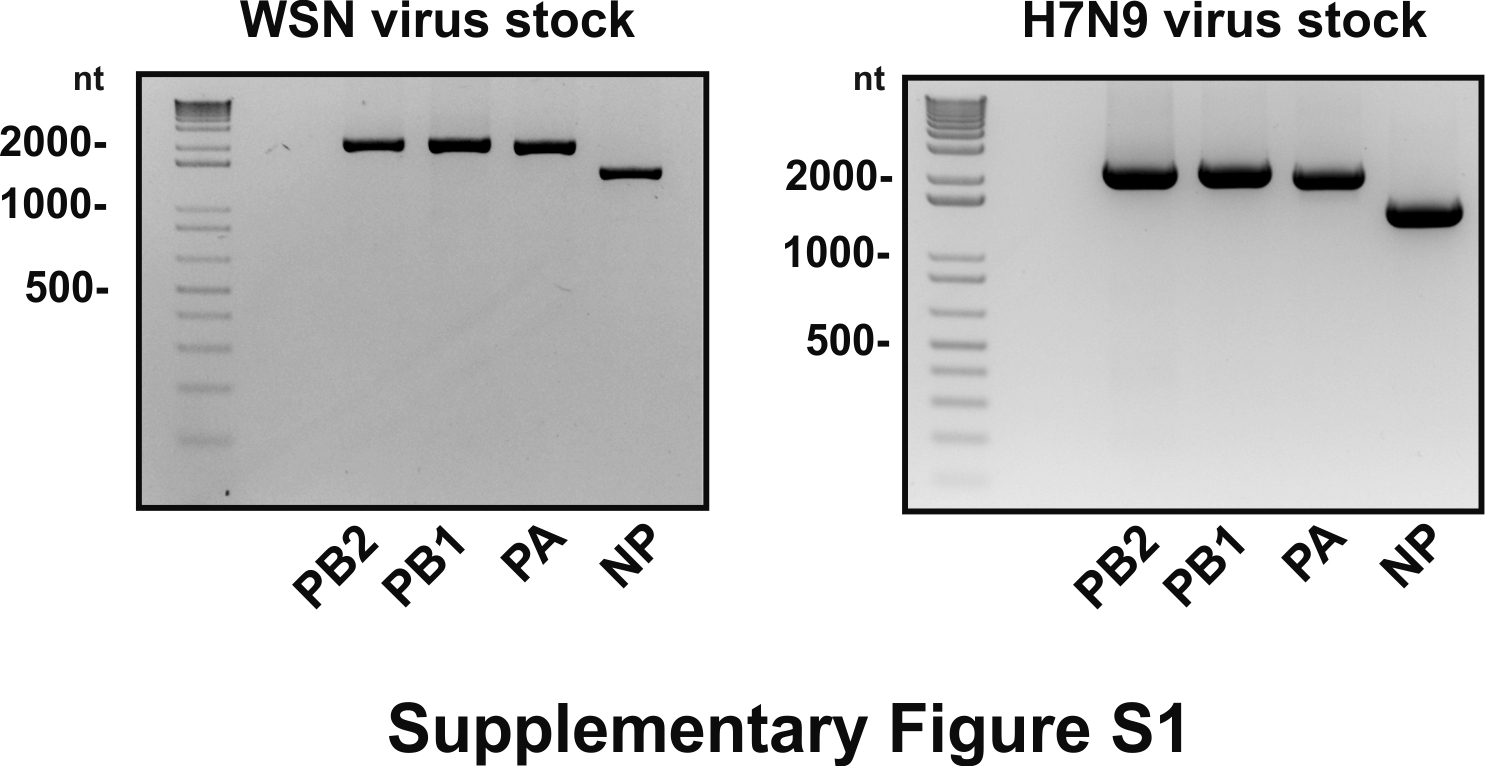

Supplement: Supplemental Material [file TEMI_A_1611346_SM4165.zip › Figure_S1.jpg]
